# Supplementary material for: A hippocampus-accumbens code guides goal-directed appetitive behavior
Source: Nat Commun. 2024 Apr 12;15:3196. doi: 10.1038/s41467-024-47361-x (PMC11015045; doi:10.1038/s41467-024-47361-x)
Supplement: Supplementary file 1 — Supplementary Information [file 41467_2024_47361_MOESM1_ESM.pdf]

Supplementary Data

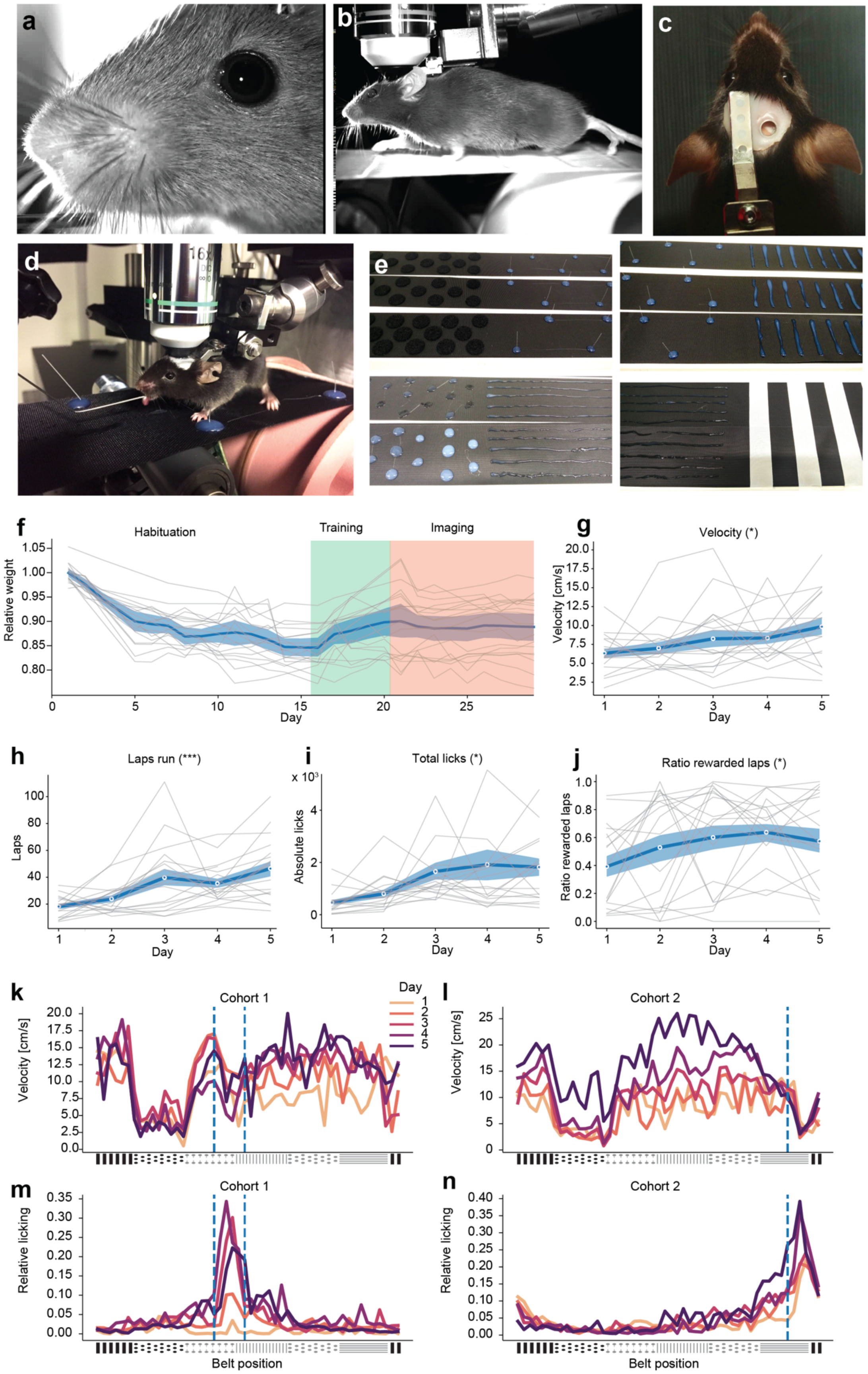

Fig. S1 | Spatial reward learning task.

a,b, Sample infrared images of face (**a**) and body (**b**) that were continuously captured at 25/75 Hz. **c**, Image of one head-fixed experimental mouse from top, illustrating craniotomy and head-fixation. **d,** Sample image of two-photon imaging setup with a head-fixed mouse on a treadmill belt, licking at the lick spout. **e,** Sample images of the six different belt texture zones, including Velcro dots, nylon spikes, vertical glue stripes, glue dots, horizontal glue stripes, white tape stripes (from left top to right bottom). **f**, Weight changes after introduction of food restriction and during training and imaging. **g-j,** Average velocity (**g**, $F(4) = 2.631$, $P = 0.0430$), number of laps run (**h**, $F(4) = 8.771$, $P < 0.001$, GG-corrected), number of licks (**i**, $F(4) = 3.883$, $P = 0.0326$, GG-corrected), and ratio of rewarded laps (**j**, $F(4) = 3.331$, $P = 0.0157$) increase over the course of five training days (all repeated-measures ANOVAs). Gray lines indicate data points of individual animals, blue shade represents SEM, blue line represents mean. **k-n**, Average velocity (**k,l**) and average licking (**m,n**) changes across belt position and days of mice tested with reward zone in “center” of belt (**k, m**) and “end” of belt (**l, n**). Blue dashed line represents presence of reward zone. $n = 18$ mice (9 in cohort 1, 9 in cohort 2). All data are presented as mean ± SEM. *$P < 0.05$, ***$P < 0.001$. Source data are provided as a Source Data file.

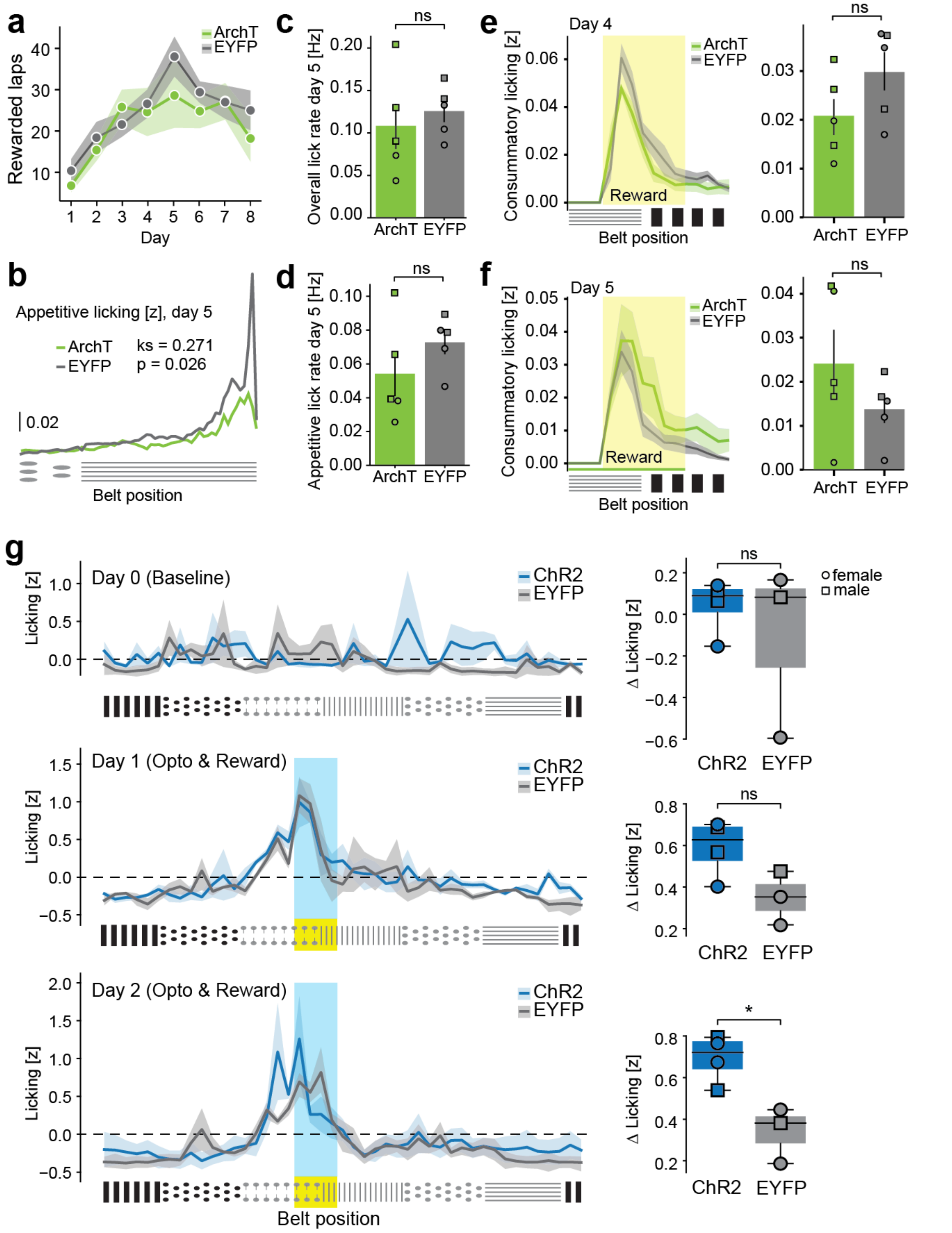

Fig. S2 | Optogenetic inhibition of dHPC→NAc pathway does not alter consummatory licking but optogenetic activation enhances spatial reward learning.

a, Number of rewarded laps is not significantly different across days between mice expressing ArchT or EYFP, all $t(8) < 1$, $P = 1$. Mixed ANOVA, main effect group $F(1, 8) = 0.754$, $P = 0.410$, main effect day $F(9, 72) = 6.47$, P (GG-corrected) < 0.001, interaction effect $F(9, 72) = 0.370$, $P =$

0.946, followed by post-hoc Bonferroni-corrected *t*-tests. **b**, Appetitive licking is differentially distributed along spatial positions between ArchT (green) and EYFP (grey) animals on day 5, 2-sample Kolmogorov-Smirnov test (ks = 0.271, P = 0.0257). Shown are spatially binned appetitive lick data, averaged across laps and mice, binned into 1 cm spatial bins from 60 cm before reward zone start up to reward zone start. **c-d**, On day 5, overall lick rate (**c**) and appetitive lick rate (**d**) are not different between ArchT (green) and EYFP (grey) animals, $t_{overall}(8) = 0.552$, P = 0.596; $t_{appetitive}(8) = 0.545$, P = 0.601. Two-tailed independent samples *t*-test. Data are presented as mean ± SEM. **e-f**, Consummatory licking does not differ between ArchT (green) and EYFP (grey) animals, neither on day 4 without light stimulation (e), nor on day 5 with light stimulation (f), $t_{overall}(8) = 1.56$, P = 0.158; $t_{appetitive}(8) = -1.25$, P = 0.247. n = 10 mice (5 ArchT, 5 EYFP). Two-tailed independent samples *t*-test. Data are presented as mean ± SEM. **g,** Optogenetic activation at reward zone leads to increased appetitive licking. Left panels show position-averaged z-scored lick activity for ChR2 (blue traces) and EYFP control (grey traces) animals, while animals received no reward nor light stimulation on the belt (top, day 0), and when animals received both light stimulation and reward at a 30 cm location on the belt (middle, bottom, days 1-2). Right panels show comparisons of baseline-corrected appetitive licking near reward zone start between ChR2 (blue) and EYFP (grey) animals. No differences are found on baseline day 0 ($t(2.31) = 0.63$, P = 0.59) nor training day 1 ($t(4.65) = 2.37$, P = 0.068), but on training day 2 ($t(4) = 3.67$, P = 0.021; all two-tailed Welch's *t*-tests), n = 7 mice (4 ChR2, 3 EYFP). Boxes represent quartiles and whiskers represent outlier-corrected minima and maxima of the distribution. Shades around traces indicate standard error of the mean (SEM); circular data points refer to female mice, square data points to male mice. ns: not significant, $*P < 0.05$. Source data are provided as a Source Data file.

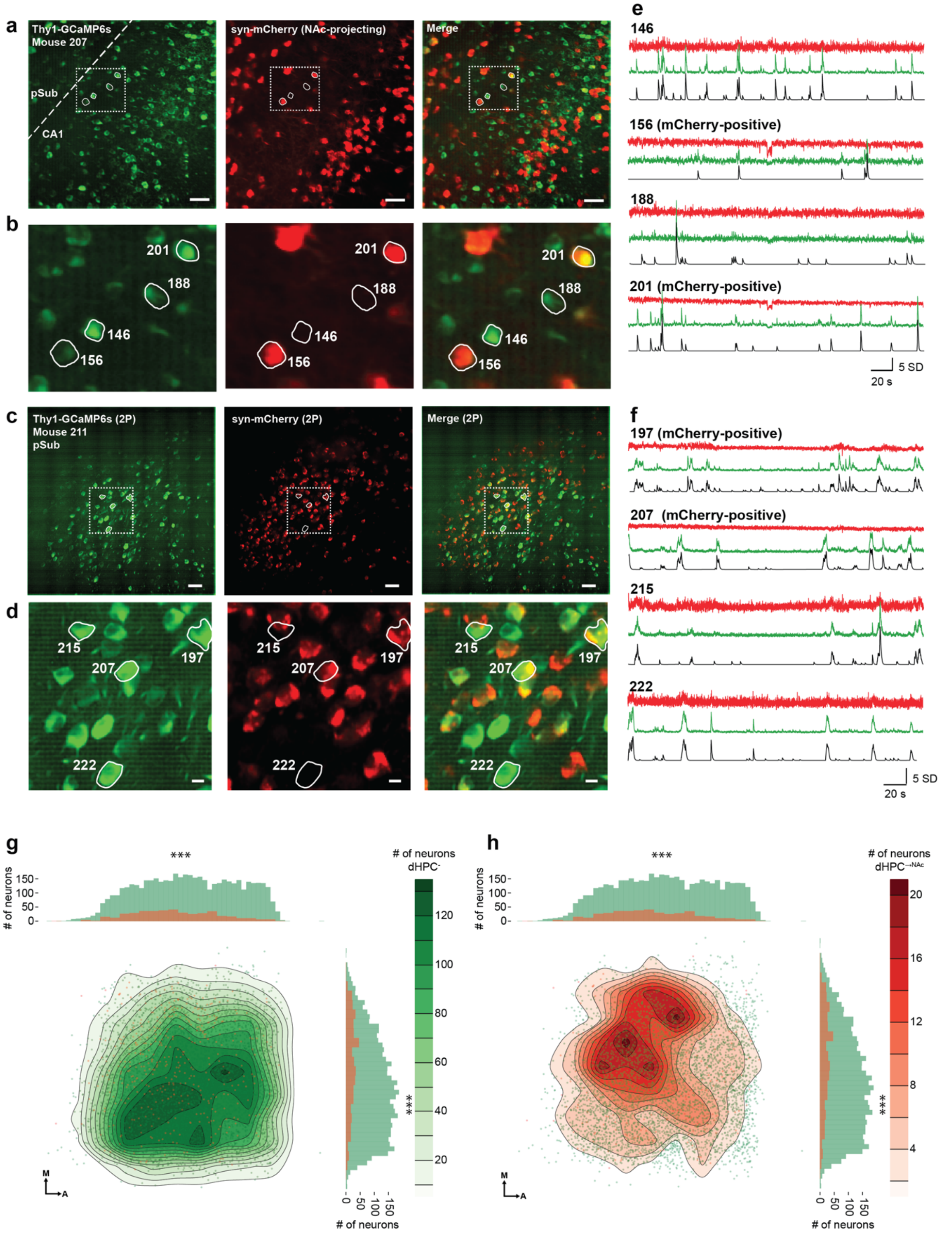

Fig. S3 | *In vivo* dual-color two-photon imaging of dynamic GCaMP and static mCherry signals.

a, Sample field of view (FOV) of one imaged region crossing putative CA1 and prosubiculum (pSub). Green channel shows GCaMP6s local correlations image, red channel shows average motion-corrected mCherry signal. **b,** Detail of region in (**a**) clearly outlining spectrally separate channels. **c,d,** Sample FOV of another region in putative prosubiculum (**c**) with detailed view (**d**). Scale bars represent 50 µm (**a,c**) or 10 µm (**b,d**). **e,f,** Z-scored fluorescent temporal sample

traces of regions of interest (ROIs) shown in (**a-d**). Red traces show raw fluorescence dynamics from imaging red channel, green traces show raw fluorescence dynamic from green channel, and black traces show denoised calcium signal. **g-h,** Relative anatomical locations of all neuronal centroids imaged along mediolateral (medial at top) and anterior-posterior (anterior at right) axes. For this, each field of view's centroids were normalized beforehand. Shown are identical histograms of both axes containing $dHPC^{-}$ (green) and $dHPC^{\rightarrow NAc}$ (red) neurons, as well as contour plots for $dHPC^{-}$ (**g**) and $dHPC^{\rightarrow NAc}$ (**h**) neurons separately. Each dot represents the normalized location of one neuron imaged during experiments ($dHPC^{-}$ green, and $dHPC^{\rightarrow NAc}$ red). The two populations were concentrated differently along both axes: $dHPC^{\rightarrow NAc}$ were more concentrated on the posterior ($t(557.7) = 6.76$, $P < 0.001$) and the medial end ($t(533.0) = 9.96$, $P < 0.001$); all two-tailed Welch's *t*-tests, $n = 5,372$ neurons. ***$P < 0.001$. Source data are provided as a Source Data file.

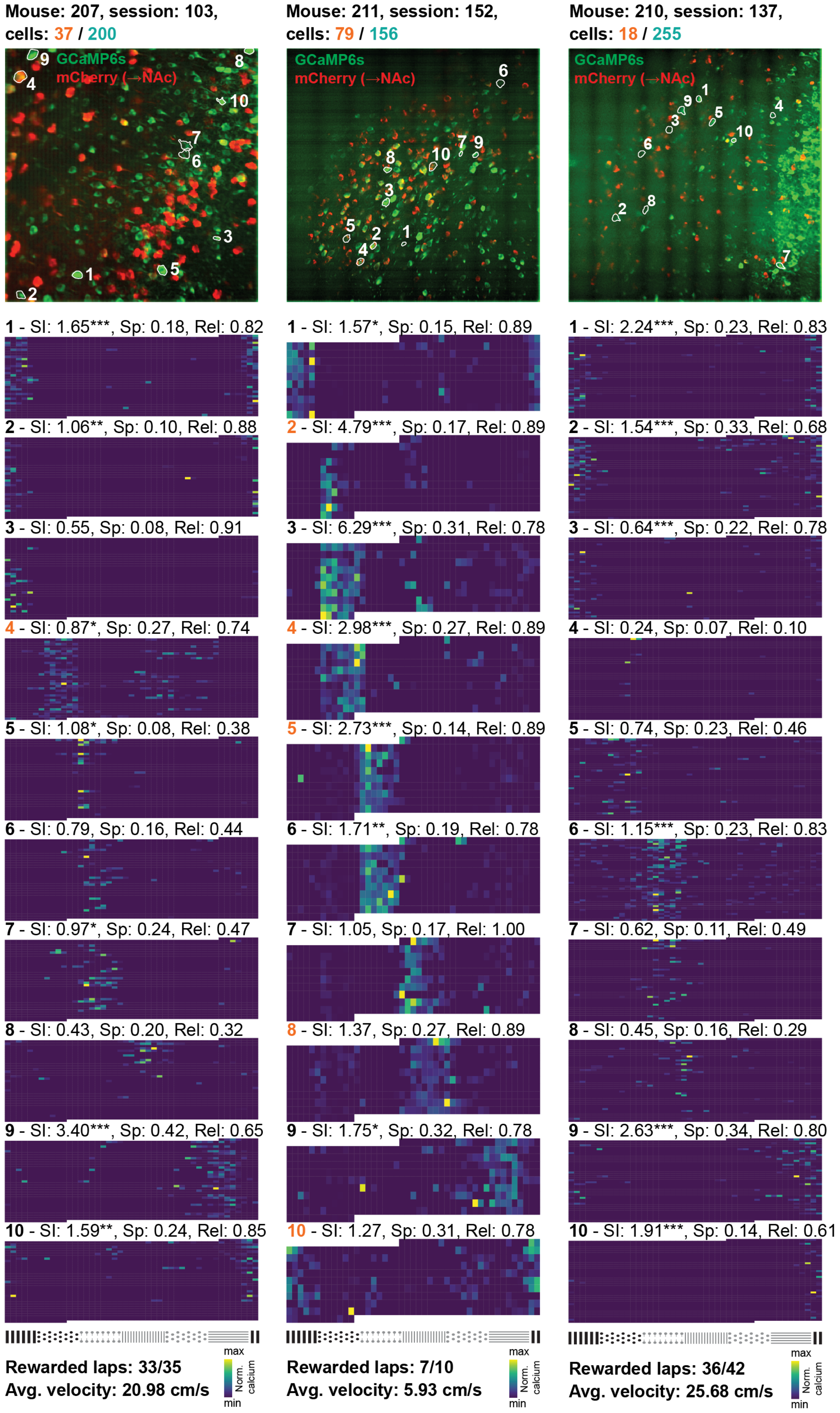
Mouse: 207, session: 103,
cells: 37 / 200
GCaMP6s
mCherry (→NAc)
1 - SI: 1.65***, Sp: 0.18, Rel: 0.82
2 - SI: 1.06**, Sp: 0.10, Rel: 0.88
3 - SI: 0.55, Sp: 0.08, Rel: 0.91
4 - SI: 0.87*, Sp: 0.27, Rel: 0.74
5 - SI: 1.08*, Sp: 0.08, Rel: 0.38
6 - SI: 0.79, Sp: 0.16, Rel: 0.44
7 - SI: 0.97*, Sp: 0.24, Rel: 0.47
8 - SI: 0.43, Sp: 0.20, Rel: 0.32
9 - SI: 3.40***, Sp: 0.42, Rel: 0.65
10 - SI: 1.59**, Sp: 0.24, Rel: 0.85
Rewarded laps: 33/35
Avg. velocity: 20.98 cm/s
Mouse: 211, session: 152,
cells: 79 / 156
GCaMP6s
mCherry (→NAc)
1 - SI: 1.57*, Sp: 0.15, Rel: 0.89
2 - SI: 4.79***, Sp: 0.17, Rel: 0.89
3 - SI: 6.29***, Sp: 0.31, Rel: 0.78
4 - SI: 2.98***, Sp: 0.27, Rel: 0.89
5 - SI: 2.73***, Sp: 0.14, Rel: 0.89
6 - SI: 1.71**, Sp: 0.19, Rel: 0.78
7 - SI: 1.05, Sp: 0.17, Rel: 1.00
8 - SI: 1.37, Sp: 0.27, Rel: 0.89
9 - SI: 1.75*, Sp: 0.32, Rel: 0.78
10 - SI: 1.27, Sp: 0.31, Rel: 0.78
Rewarded laps: 7/10
Avg. velocity: 5.93 cm/s
Mouse: 210, session: 137,
cells: 18 / 255
GCaMP6s
mCherry (→NAc)
1 - SI: 2.24***, Sp: 0.23, Rel: 0.83
2 - SI: 1.54***, Sp: 0.33, Rel: 0.68
3 - SI: 0.64***, Sp: 0.22, Rel: 0.78
4 - SI: 0.24, Sp: 0.07, Rel: 0.10
5 - SI: 0.74, Sp: 0.23, Rel: 0.46
6 - SI: 1.15***, Sp: 0.23, Rel: 0.83
7 - SI: 0.62, Sp: 0.11, Rel: 0.49
8 - SI: 0.45, Sp: 0.16, Rel: 0.29
9 - SI: 2.63***, Sp: 0.34, Rel: 0.80
10 - SI: 1.91***, Sp: 0.14, Rel: 0.61
Rewarded laps: 36/42
Avg. velocity: 25.68 cm/s
max
Norm. calcium
min

Fig. S4 | Spatial tuning of different neurons in sample FOVs.

Three representative fields of view (FOVs) from three different mice are shown, with single-lap spatial calcium activity of each 10 representative neurons, some putatively NAc-projecting. FOV information is shown on top, including numbers of identified $dHPC^{-}$ (green) and $dHPC^{\rightarrow NAc}$ (red) neurons. FOVs shown are composites of CaImAn maximum local correlation images (*caiman.summary_images.max_correlation_image*) of GCaMP channel (green) and the averaged motion-corrected red channel with NAc-projecting mCherry fluorescence. Contours of ten representative neurons from each FOV are indicated with white outlines and numbers that refer to spatially averaged calcium activity below. Each neuron's normalized average calcium activity across 45 spatial bins per lap (y axis) is shown with key spatial information values above (SI: spatial information (Skaggs et al., 1993), Sp: Sparsity (Jung et al., 1994), Rel: reliability of each neuron's per-lap maximum activity to occur within the place field). Asterisks refer to significant spatial modulation (classified place cells) according to a 1,000x randomly shuffled distribution (* > 95th percentile; ** > 99th percentile; *** > 99.9th percentile). Orange numbers refer to NAc-projecting neurons. Source data are provided as a Source Data file.

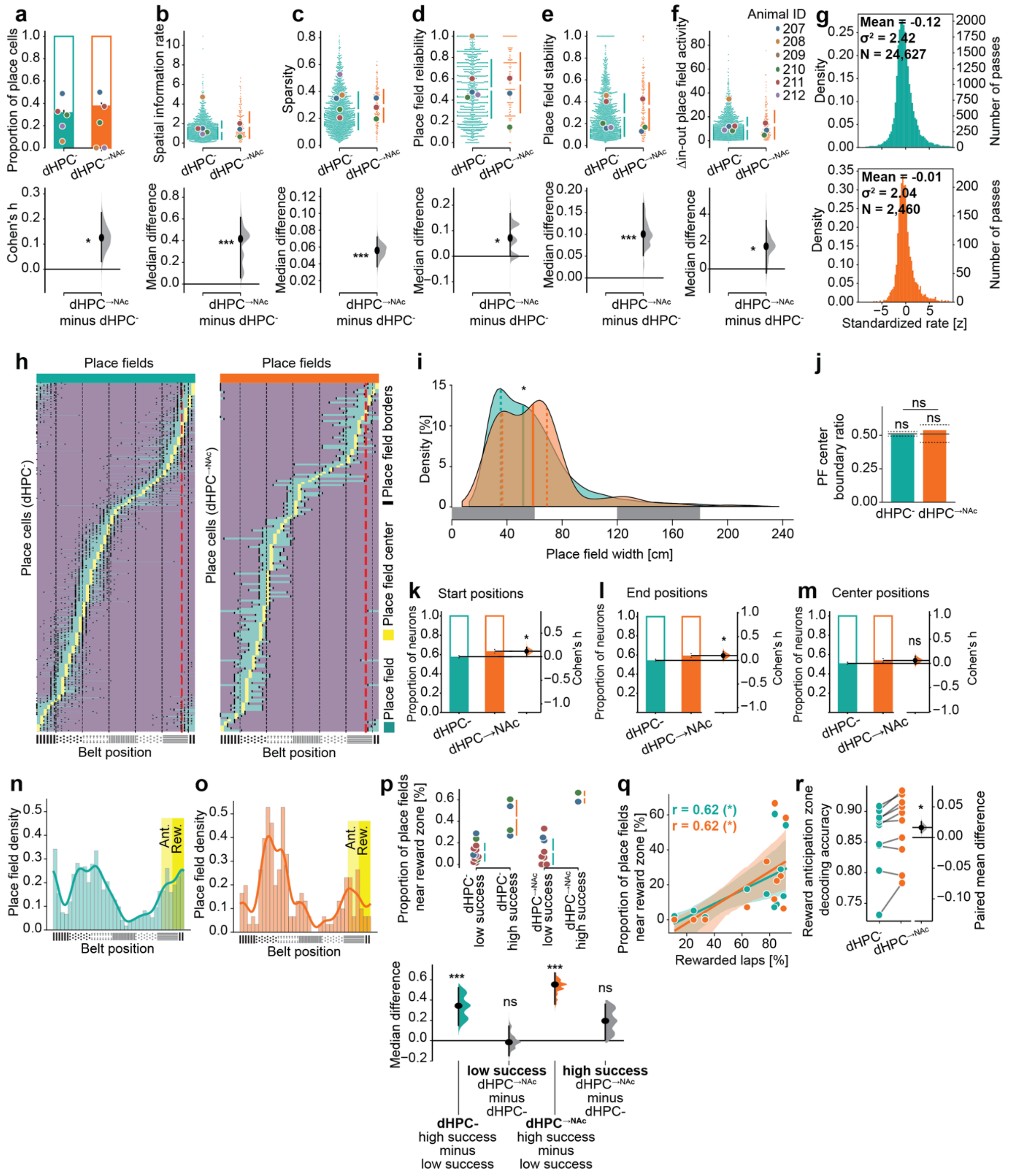

Fig. S5 | Spatial tuning and place field distribution of projection-specific dHPC neurons.

a-f, Estimation statistics of data shown in Fig. 3f-k. **a**, Place cell proportion is higher in dHPC→NAc (red) than in dHPC- (green) populations, Cohen's h = 0.125 (95% confidence intervals: 0.030, 0.226), *P* = 0.012, *n* = 5372 neurons from 5 mice. **b**, Spatial information rate is higher in dHPC→NAc (red) than in dHPC- (green) populations, median difference = 0.416 (95% confidence intervals: 0.057, 0.615), *P* < 0.0001. **c**, Sparsity is higher in dHPC→NAc (red) than in dHPC- (green) populations, median difference = 0.056 (95% confidence intervals: 0.036, 0.072), *P* = 0.0002. **d**, Place field reliability is higher in dHPC→NAc (red) than in dHPC- (green) populations, median difference = 0.0714 (95% confidence intervals: 0.0, 0.167), *P* = 0.015. **e**, Place field stability is

higher in $dHPC^{\rightarrow NAc}$ (red) than in $dHPC^{-}$ (green) populations, median difference = 0.101 (95% confidence intervals: 0.051, 0.171), $P < 0.0001$. **f**, Relative in-place field activity is higher in $dHPC^{\rightarrow NAc}$ (red) than in $dHPC^{-}$ (green) populations, median difference = 1.650 (95% confidence intervals: -0.302, 3.545), $P = 0.022$. **g**, Histograms showing the distribution of the standardized calcium activity presented for $dHPC^{\rightarrow NAc}$ (red) and $dHPC^{-}$ (green) populations. $dHPC^{-}$ neurons show increased overdispersion compared to $dHPC^{\rightarrow NAc}$ neurons ($\sigma^2 = 2.42$, N=24,627 passes vs. $\sigma^2 = 2.04$, N=2,460 passes, $P < 0.001$, Levene test for equal variances. **h**, Extent of place fields of $dHPC^{-}$ (left) and $dHPC^{\rightarrow NAc}$ (right) place cells, sorted by each place cell's center of mass (COM). Turquoise represents place field, yellow represents COM, black lines represent left and right borders of place fields. Red dashed line represents beginning of reward zone, black dashed lines represent belt texture borders. **i**, Width of place fields is unevenly distributed between $dHPC^{-}$ (green; quartiles 35.6 cm / 51.7 cm / 69.0 cm) and $dHPC^{\rightarrow NAc}$ (red; quartiles 36.5 cm / 58.9 cm / 69.0 cm) place cells (Kolmogorov-Smirnov test, $D = 0.116$, $P = 0.0299$). Gray bars represent belt texture zones (60 cm). **j,** Place field centers are not biased with respect to texture boundaries (percentile < 95th, permutation test) and the ratio is not different between neuronal populations ($\chi^2(1, 5372) = 1.646$, $P = 0.1995$). **k-m**, Estimation statistics of data shown in Figs. 4c, d and S5j. **k**, Place field start positions of $dHPC^{\rightarrow NAc}$ place fields are significantly overrepresented compared to the $dHPC^{-}$ population, Cohen's $h = 0.116$ (95% confidence intervals: 0.019, 0.212), $P = 0.0152$. **l**, Place field end positions of $dHPC^{\rightarrow NAc}$ place fields are significantly overrepresented compared to the $dHPC^{-}$ population, Cohen's $h = 0.101$ (95% confidence intervals: 0.002, 0.198), $P = 0.0422$. **m**, Place field center positions of $dHPC^{\rightarrow NAc}$ place fields are not significantly different compared to the $dHPC^{-}$ population, Cohen's h = 0.060 (95% confidence intervals: -0.038, 0.158), $P = 0.215$. **n, o**, Place field density between reward and anticipation zones compared to the rest of the belt for $dHPC^{-}$ (**n**) and $dHPC^{\rightarrow NAc}$ (**o**) populations. **p,** Estimation statistics of data shown in Figs. 4f. High-success trials show a higher proportion of place fields near reward zone for $dHPC^{-}$ neurons (median difference = 0.344, 95% confidence intervals: 0.151, 0.521, $P < 0.0001$) and for $dHPC^{\rightarrow NAc}$ neurons (median difference = 0.554, 95% confidence intervals: 0.361, 0.667, $P < 0.0001$). Proportion of place fields near reward zone were not different between $dHPC^{\rightarrow NAc}$ and $dHPC^{-}$ populations for high-success trials (median difference = 0.195, 95% confidence intervals: 0.010, 0.358, $P = 0.136$) or for low-success trials (median difference = -0.0144, 95% confidence intervals: -0.147, 0.142, $P = 0.704$), $n = 16$ imaging sessions. **q,** Reward and anticipation zone overrepresentation correlates with behavioral success (percentage of rewarded laps per session) for both $dHPC^{-}$ (green) and $dHPC^{\rightarrow NAc}$ (red) populations (Pearson correlation; $r_{dHPC-}(15) = 0.622$, $P = 0.0107$; $r_{dHPC\rightarrow NAc}(10) = 0.619$, $P = 0.0421$; $n = 11$ imaging sessions). **r,** Estimation statistics of data shown in Figs. 4g. Reward anticipation zone decoding accuracy is higher in $dHPC^{\rightarrow NAc}$ neurons compared to $dHPC^{-}$ neurons. Mean difference = 0.0163 (95% confidence intervals: 0.007, 0.027), $P = 0.0176$, $n = 10$ imaging sessions. Colored dots represent mean values per animal from which neurons were recorded. All estimation plots consist of the following: upper/left panels show raw data points representing either one cell/animal/recording, with the gapped lines on the right as median (gap) ± s.d. (vertical ends); lower/right panels show the respective differences (Δ) computed from 5,000 bootstrapped resamples (black dot, mean; black ticks, 95% confidence interval; filled curve, sampling-error distribution). *n* (**b-f, h, i, n, o**) = 1750 place cells from 5 mice; *n* (**j-m**) = 5372 place cells from 6 mice; ns: not significant, $^{*}P < 0.05$, $^{***}P < 0.001$. Source data are provided as a Source Data file.

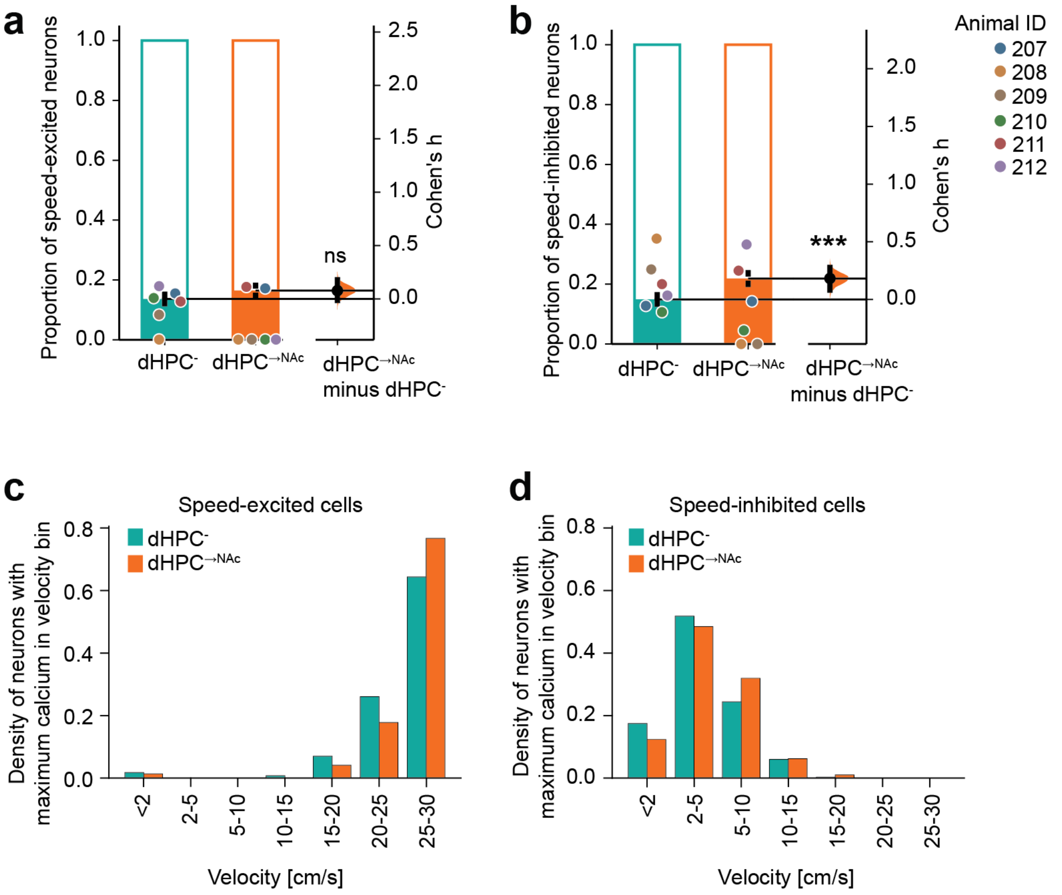

Fig. S6 | dHPC$^{\rightarrow NAc}$ neurons are more negatively velocity-modulated than dHPC^{-} neurons.

a-b, Estimation statistics of Figs. 5d, h. Gardner-Altman plots showing the proportions of speed-excited (**a**) or speed-inhibited (**b**) neurons for dHPC^{-} (green) and dHPC$^{\rightarrow NAc}$ (red) neurons; vertical bars represent s.d.; right panels show Cohen's h computed from 5,000 bootstrapped resamples (black dot, mean; black ticks, 95% confidence interval; filled curve, sampling-error distribution). Colored dots represent proportion values per animal. **a**, Speed-excited neurons are similarly distributed between dHPC^{-} and dHPC$^{\rightarrow NAc}$ populations, Cohen's h = 0.0791 (95% confidence intervals: -0.0129, 0.1798), P = 0.0934. **b,** Speed-inhibited neurons are overrepresented in dHPC$^{\rightarrow NAc}$ neurons compared to dHPC^{-} neurons, Cohen's h = 0.181 (95% confidence intervals: 0.0814, 0.277), P = 0.0004. **c-d**, Distribution of calcium maximum speed bins of speed-excited (**c**) and speed-inhibited (**d**) neurons. Proportions are not different between dHPC^{-} and dHPC$^{\rightarrow NAc}$ populations for speed-excited neurons (χ^2(4, 830) = 4.799, P = 0.309) and speed-inhibited neurons (χ^2(4, 744) = 4.740, P = 0.315). All n = 5372 neurons from 6 mice. ns: not significant, ***P < 0.001. Source data are provided as a Source Data file.

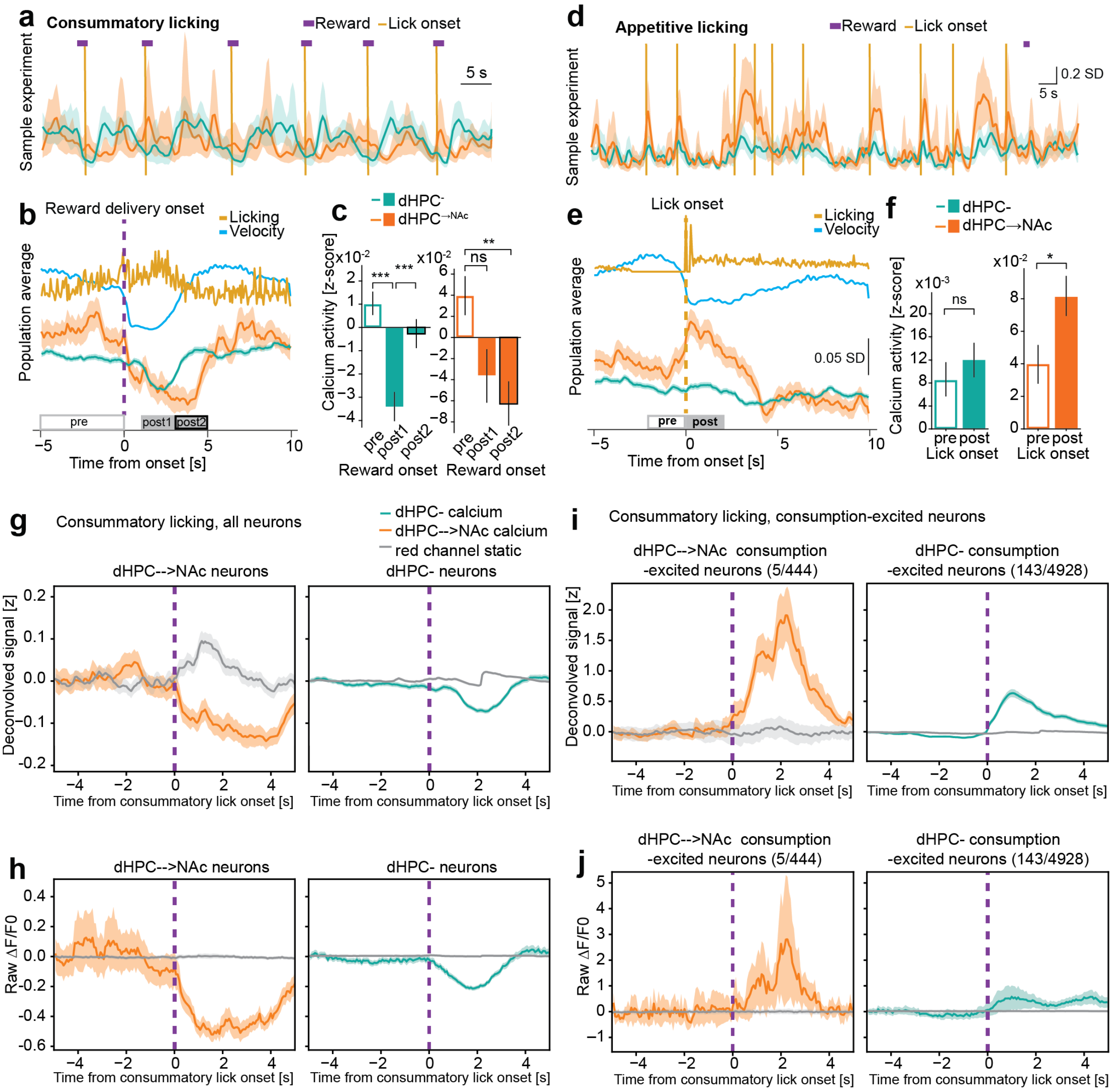

Fig. S7 | Consummatory licking results in negative calcium responses, appetitive licking results in a dHPC$^{\rightarrow NAc}$-specific calcium increase.

a-c, Consummatory licking leads to a depression in neural activity. **a,** Sample experiment showing reward dispensation (purple bars) and consummatory licking onsets (golden vertical lines) and population calcium activity from dHPC^{-} (green) and dHPC$^{\rightarrow NAc}$ (red) neurons. Note the robust depression of neural activity during times of reward consumption. **b,** Event-triggered average traces around reward delivery onset, including licking and speed as well as population average calcium activity of dHPC^{-} (green) and dHPC$^{\rightarrow NAc}$ (red) neurons. Gray rectangles indicate time windows for comparing calcium activity shown in (**c**). **c,** Calcium activity is differentially modulated by reward onset (two-way mixed ANOVA; $F_{reward\_timing}(2, 10740) = 19.380$, P(GG-corrected) < 0.001, $F_{projection}(1, 5370) = 0.798$, $P = 0.372$, $F_{interaction}(2, 10740) = 5.559$, $P = 0.0039$). Post-hoc pairwise t-tests with Bonferroni correction were performed for each interaction and the results indicated with asterisks. **d-f,** Appetitive licking is accompanied by increased neural activity in dHPC$^{\rightarrow NAc}$ neurons but not dHPC^{-} neurons. **d,** Representative example showing reward dispensation (purple bar) and appetitive licking onsets (golden vertical lines) and population calcium activity from dHPC^{-} (green) and dHPC$^{\rightarrow NAc}$ (red) neurons. Note the robust increase of

neural activity around lick onsets in the $dHPC^{\rightarrow NAc}$ population. **e,** Event-triggered average traces around appetitive licking onset, including licking and speed as well as population average calcium activity of $dHPC^{-}$ (green) and $dHPC^{\rightarrow NAc}$ (red) neurons. Gray rectangles indicate time windows for comparing calcium activity shown in (**f**). **f,** Calcium activity is differentially modulated by appetitive licking onset only in $dHPC^{\rightarrow NAc}$ neurons; two-way mixed ANOVA; $F_{lick\_timing}(1, 5370) = 2.843$, $P = 0.0918$, $F_{projection}(1, 5370) = 43.779$, $P < 0.001$, $F_{interaction}(1, 5370) = 7.073$, $P = 0.0079$. Post-hoc *t*-tests with Bonferroni correction: $t_{dHPC-}(4927) = 0.871$, $P = 0.768$; $t_{dHPC\rightarrow NAc}(443) = 2.470$, $P = 0.0277$. **g-j,** Event-triggered average traces around consummatory lick onsets for all neurons (**g-h**) and consumption-excited neurons (**i-j**). Calcium (red/green) and static (grey) signals from $dHPC^{\rightarrow NAc}$ (red) and $dHPC^{-}$ (green). Traces in **g, i** show z-scored deconvolved signals (using OASIS), while traces in **h, j** show raw ΔF/F0 signals (F0 defined as 8th percentile of trace). Excited/inhibited neurons were defined as described in Methods: Wilcoxon signed-rank tests for each neuron's average signal -2:0 s prior to event were compared to the average signal 0:2 s after event. Significant and positively modulated neurons are termed "excited"; significant and negatively modulated neurons are termed "inhibited". Data in **b, c, e-h** are from n = 5372 neurons from 6 mice; data in **i, j** are from *n* = 148 neurons from 3 mice. All data are presented as mean ± SEM. ns: not significant, $^{**}P < 0.01$, $^{***}P < 0.001$. Source data are provided as a Source Data file.

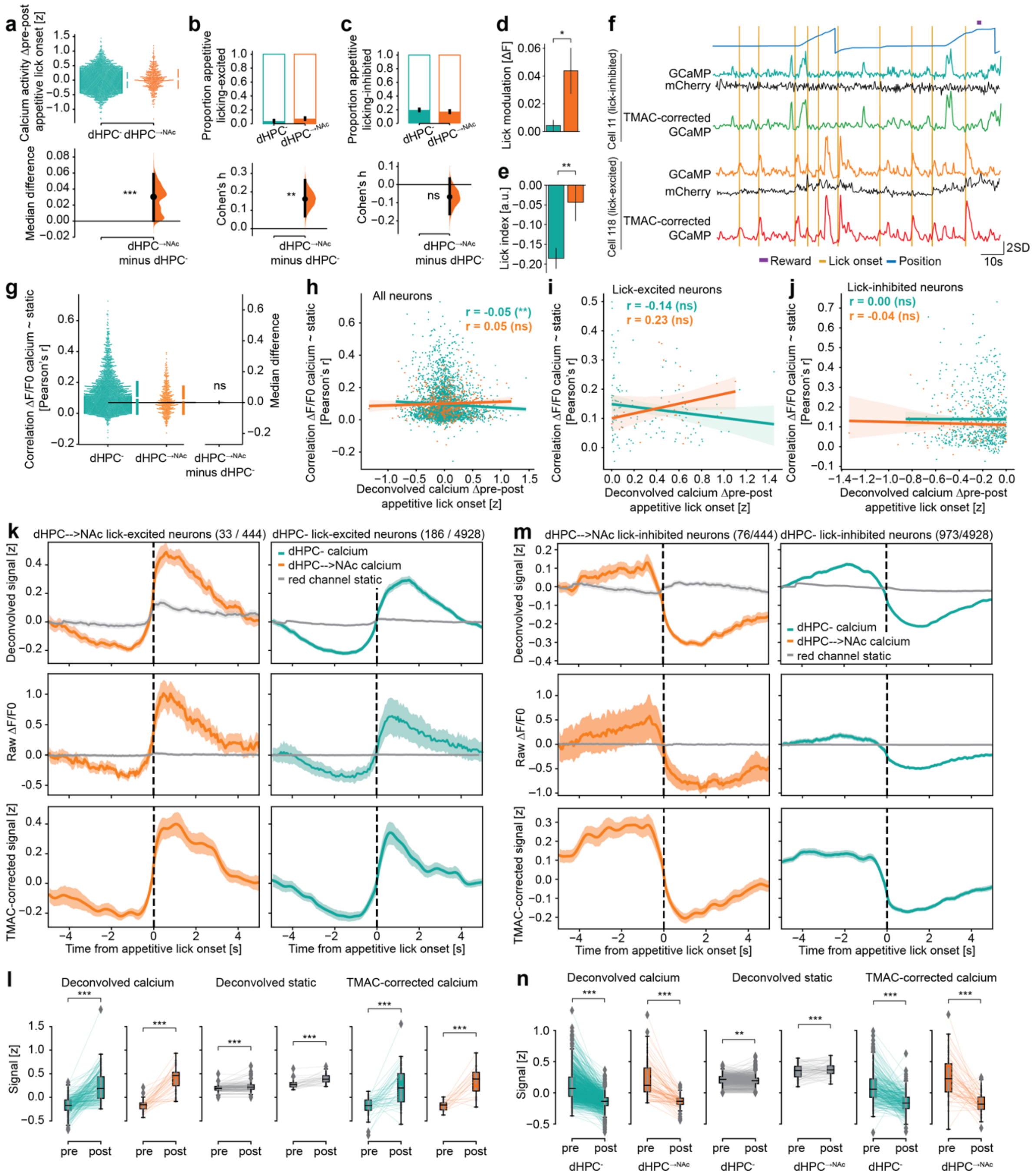

Fig. S8 | Appetitive lick modulation cannot be explained by changes in static channel.

a-c, Estimation statistics of Figs. 6 e, f and Fig. S8d. Cumming estimation plots showing the relative calcium activity differences pre-post appetitive lick onset (**a**), proportions of appetitive lick-excited (**b**) or appetitive lick-inhibited (**c**) neurons for dHPC^{-} (green) and dHPC$^{\rightarrow NAc}$ (red) neurons; vertical bars represent s.d.; bottom panels show median difference (**a**) or Cohen's h (**b,c**) computed from 5,000 bootstrapped resamples (black dot, me(di)an; black ticks, 95% confidence interval; filled curve, sampling-error distribution). **a,** dHPC$^{\rightarrow NAc}$ neurons' calcium activity is more strongly modulated by appetitive lick onset than dHPC^{-} neurons, median difference = 0.0303 (95% confidence intervals: 0.0005, 0.0586), *P* < 0.0001. **b,** A larger proportion of dHPC$^{\rightarrow NAc}$ neurons is lick-excited compared to dHPC^{-} neurons, Cohen's h = 0.161

(95% confidence intervals: 0.0682, 0.263), *P* = 0.0018. **c,** The proportions of lick-inhibited neurons are not different between dHPC$^{\rightarrow NAc}$ and dHPC^{-} neurons, Cohen's h = - 0.0678 (95% confidence intervals: - 0.1624, 0.0334), *P* = 0.173. **d,** Lick modulation (average of each trial's pre-post difference) is significantly increased in dHPC$^{\rightarrow NAc}$ neurons (Welch's *t*(496.11) = 2.379, *P* = 0.0177). **e,** Lick index (average calcium activity difference during licking) is significantly increased in dHPC$^{\rightarrow NAc}$ neurons (Welch's *t*(454.19) = 2.729, *P* = 0.0066). **f,** Sample behavioral and calcium traces as shown in Fig. 5a, including average static red channel's signal for both cells' spatial footprints (black traces underneath respective calcium trace) and TMAC-corrected calcium traces. Calcium traces shown are z-scored ΔF/F0 values. TMAC correction is based on these traces. **g**, Denoised calcium activity correlates similarly strongly with static signals between dHPC- (green) and dHPC→NAc (red) populations, median difference = 0.00091 (95% confidence intervals: -0.0075, 0.0100), P = 0.775, *P*(Kruskal-Wallis) = 0.109. Cumming estimation plot showing each neuron's Pearson's correlation r value from its denoised calcium trace to its static red signal; vertical bars represent s.d.; bottom panels show median difference computed from 5,000 bootstrapped resamples (black dot, me(di)an; black ticks, 95% confidence interval; filled curve, sampling-error distribution). **h-j,** Scatter plots of appetitive lick calcium response magnitude (x-axis) and correlation values between ΔF/F0 calcium activity and red static channel (y-axis) for all dHPC$^{\rightarrow NAc}$ (red) and dHPC^{-} (green) neurons (**h**), only lick-excited neurons (**i**), and only lick-inhibited neurons (**j**), including line of best fit. Correlations between static channel correlations and appetitive lick magnitude were weakly but significantly different for all dHPC^{-} neurons (*r*(3249) = -0.05, *P* = 0.0027) but not significant for all dHPC$^{\rightarrow NAc}$ neurons (*r*(419) = 0.05, *P* = 0.33), nor for dHPC^{-} lick-excited neurons (*r*(124) = -0.145, *P* = 0.109) nor dHPC$^{\rightarrow NAc}$ lick-excited neurons (*r*(28) = 0.239, *P* = 0.220), nor for dHPC^{-} lick-inhibited neurons (*r*(699) = 0.00, *P* = 0.979) nor dHPC$^{\rightarrow NAc}$ lick-inhibited neurons (*r*(69) = -0.042, *P* = 0.732). **k-n,** Event-triggered average traces of lick-excited (**k, l**) and lick-inhibited (**m, n**) neurons around appetitive licking onset, as in Fig. 6c, d, including average static red signal (grey) for dHPC^{-} (green) and dHPC$^{\rightarrow NAc}$ (red) populations. Raw ΔF/F0 signals (F0 defined as 8th percentile of trace) are shown below deconvolved traces. TMAC-corrected signals were processed applying Two-channel Motion Artifact Correction (TMAC)(Creamer et al., 2022) on z-scored ΔF/F0 calcium activity before event averaging. **k**, Traces of lick-excited neurons from -5 to +5 seconds around appetitive lick onset. **l**, Deconvolved calcium activity differs between -2:0 seconds and 0:2 seconds around the onset of appetitive licking in lick-excited dHPC$^{\rightarrow NAc}$ (*t*(32) = 11.401, *P* < 0.001) and dHPC^{-} (*t*(185) = 15.504, *P* < 0.001) neurons. Mixed ANOVA, main effect projection F(1, 217) = 5.675, *P* = 0.018, main effect pre/post F(1, 217) = 331.92, *P* < 0.001, interaction effect F(1, 217) = 3.941, *P* = 0.0484, followed by post-hoc Bonferroni-corrected *t*-tests. Deconvolved static mCherry signal differs between -2:0 seconds and 0:2 seconds around the onset of appetitive licking in lick-excited dHPC$^{\rightarrow NAc}$ (*t*(27) = 5.392, *P* < 0.001) and dHPC^{-} (*t*(123) = 5.225, *P* < 0.001) neurons. Mixed ANOVA, main effect projection F(1, 150) = 57.98, *P* < 0.001, main effect pre/post F(1, 150) = 55.353, *P* < 0.001, interaction effect F(1, 150) = 23.87, *P* < 0.001, followed by post-hoc Bonferroni-corrected *t*-tests. TMAC-corrected calcium activity differs between -2:0 seconds and 0:2 seconds around the onset of appetitive licking in lick-excited dHPC$^{\rightarrow NAc}$ (*t*(19) = 6.966, *P* < 0.001) and dHPC^{-} (*t*(46) = 6.066, *P* < 0.001) neurons. Mixed ANOVA, main effect projection F(1, 65) = 1.194, *P* = 0.279, main effect pre/post F(1, 65) = 69.91, *P* < 0.001, interaction effect F(1, 65) = 0.540, *P* = 0.465, followed by post-hoc Bonferroni-corrected *t*-tests. **m**, Traces of lick-inhibited neurons from -5 to +5 seconds around appetitive lick onset. **n**,

Deconvolved calcium activity differs between -2:0 seconds and 0:2 seconds around the onset of appetitive licking in lick-inhibited dHPC$^{\rightarrow NAc}$ (t(75) = -11.45, P < 0.001) and dHPC^{-} (t(972) = -36.29, P < 0.001) neurons. Mixed ANOVA, main effect projection F(1, 1047) = 6.849, P = 0.009, main effect pre/post F(1, 1047) = 1448.07, P < 0.001, interaction effect F(1, 1047) = 12.87, P < 0.001, followed by post-hoc Bonferroni-corrected t-tests. Deconvolved static mCherry signal differs between -2:0 seconds and 0:2 seconds around the onset of appetitive licking in lick-inhibited dHPC$^{\rightarrow NAc}$ (t(68) = 3.020, P = 0.00711) and dHPC^{-} (t(697) = -6.357, P < 0.001) neurons. Mixed ANOVA, main effect projection F(1, 765) = 261.56, P < 0.001, main effect pre/post F(1, 765) = 15.74, P < 0.001, interaction effect F(1, 765) = 43.31, P < 0.001, followed by post-hoc Bonferroni-corrected t-tests. TMAC-corrected calcium activity differs between -2:0 seconds and 0:2 seconds around the onset of appetitive licking in lick- inhibited dHPC$^{\rightarrow NAc}$ (t(57) = -8.33, P < 0.001) and dHPC^{-} (t(253) = -14.30, P < 0.001) neurons. Mixed ANOVA, main effect projection F(1, 310) = 9.72, P = 0.002, main effect pre/post F(1, 310) = 273.75, P < 0.001, interaction effect F(1, 310) = 14.03, P < 0.001, followed by post-hoc Bonferroni-corrected t-tests. Box-and-whisker plots in **l, n** show quartiles represented by the box and outlier-corrected minima and maxima by the whiskers. All data are presented as mean ± SEM if not described otherwise. ns: not significant, *P < 0.05, **P < 0.01, ***P < 0.001. Source data are provided as a Source Data file.

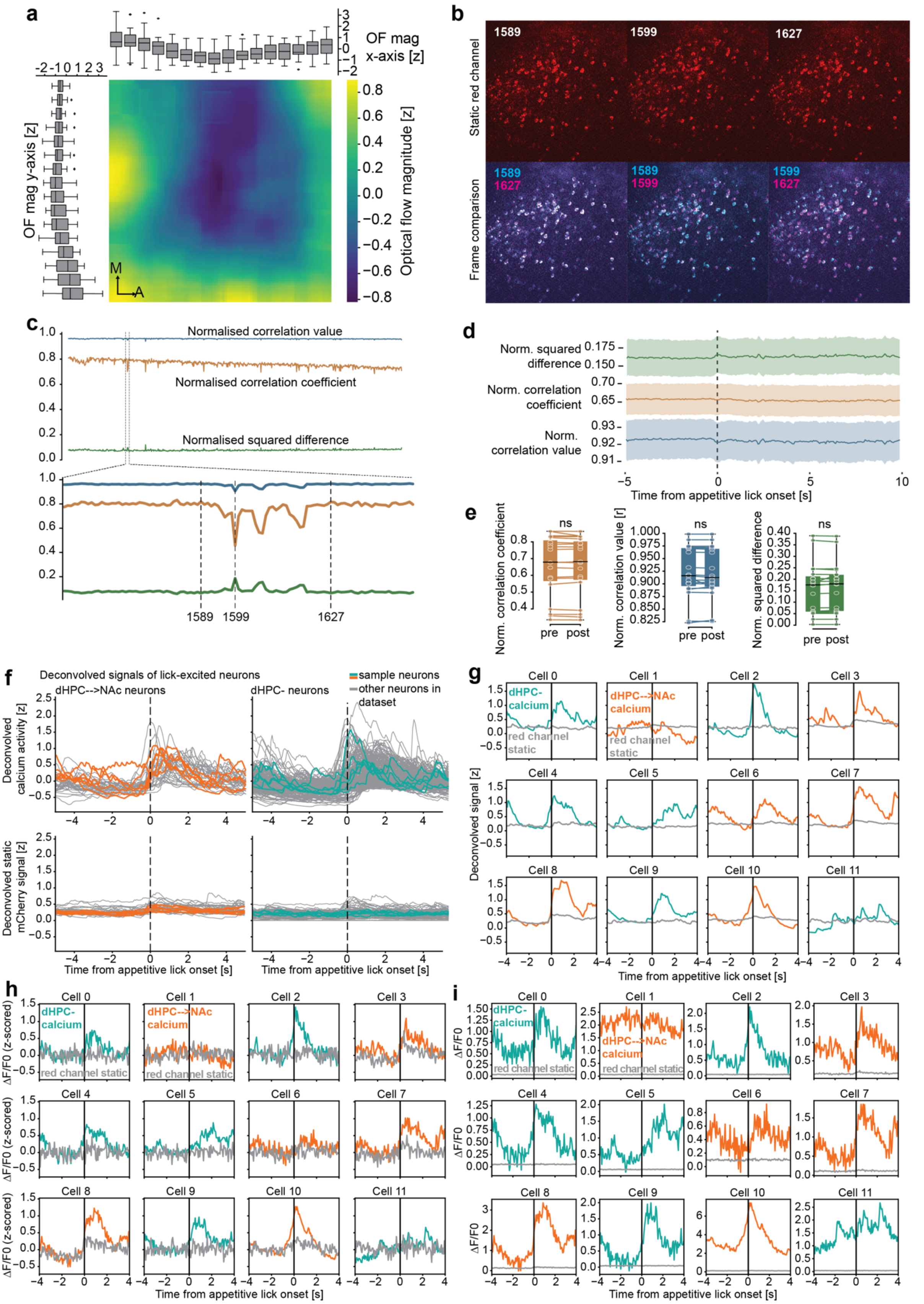
a
OF mag x-axis [z]
OF mag y-axis [z]
Optical flow magnitude [z]
M
A
b
Static red channel
Frame comparison
1589
1599
1627
c
Normalised correlation value
Normalised correlation coefficient
Normalised squared difference
1589
1599
1627
d
Norm. squared difference
Norm. correlation coefficient
Norm. correlation value
Time from appetitive lick onset [s]
e
ns
Norm. correlation coefficient
Norm. correlation value [r]
Norm. squared difference
pre
post
f
Deconvolved signals of lick-excited neurons
dHPC-->NAc neurons
dHPC- neurons
sample neurons
other neurons in dataset
Deconvolved calcium activity [z]
Deconvolved static mCherry signal [z]
Time from appetitive lick onset [s]
g
Cell 0
Cell 1
Cell 2
Cell 3
Cell 4
Cell 5
Cell 6
Cell 7
Cell 8
Cell 9
Cell 10
Cell 11
dHPC- calcium
dHPC-->NAc calcium
red channel static
Deconvolved signal [z]
Time from appetitive lick onset [s]
h
ΔF/F0 (z-scored)
dHPC- calcium
dHPC-->NAc calcium
red channel static
Time from appetitive lick onset [s]
i
ΔF/F0
dHPC- calcium
dHPC-->NAc calcium
red channel static
Time from appetitive lick onset [s]

Fig. S9 | Residual motion estimates are not lick-modulated.

a, Residual motion estimate optical flow magnitude averaged per field of view recorded. Left and top show boxplots of average values per FOV and respective xy position. Main image shows color-mapped average optical flow magnitude across all FOVs. Note that anterior, posterior, and lateral edges show higher average optical flow magnitude. **b, c**, Estimating red static channel residual motion. **b,** Three sample frames of red static channel from the same recording at time points 1589, 1599, and 1627, affected by residual motion. Lower panel shows a significant motion event around frame 1599, affecting frame comparisons between 1589 to 1599 and 1599 to 1627 but not between 1589 to 1627. **c,** Quantifying residual motion by OpenCV's template matching function resulting in values for normalised correlation value (blue), normalised correlation value (brown), and normalised squared difference (green). Upper panel shows relative stability of values over the course of a 5-minute (9,000 frames) recording. Lower panel shows a close-up view of frames 1550 to 1650, with residual motion indicated by all three measures at frame 1599. **d, e**, Residual motion is not affected by appetitive licking. **d,** Event-triggered average traces around appetitive licking onset as in (**i**) for three residual motion estimates. **e,** Normalised correlation coefficients are not significantly different before and after appetitive lick onset, $t(18) = 1.067$, $P = 0.300$, paired *t*-test. Normalised correlation values are not significantly different before and after appetitive lick onset, $t(18) = 0.9343$, $P = 0.3625$, paired *t*-test. Normalised squared differences are not significantly different before and after appetitive lick onset, $t(18) = 1.092$, $P = 0.2891$, paired *t*-test. Box-and-whisker plots show quartiles represented by the box and outlier-corrected minima and maxima by the whiskers. **f,** Averaged deconvolved and z-scored signal of lick-excited neurons around the time of appetitive lick onset. Left panels show $dHPC^{\rightarrow NAc}$ neurons, right panels show $dHPC^{-}$ neurons. Top panels show deconvolved calcium activity, bottom panels show deconvolved static mCherry signal. Coloured traces represent those neurons highlighted in Supplementary Video 3. **g-i**, Averaged signal of 12 sample cells highlighted in Supplementary Video 3, around the time of appetitive lick onset. **g,** Deconvolved signal, z-scored. **h,** Raw ΔF/F0 signal, z-scored. **i,** Raw ΔF/F0 signal (F0 defined as 8th percentile). Red traces refer to calcium signals from $dHPC^{\rightarrow NAc}$ neurons, green traces to calcium signals from $dHPC^{-}$ neurons, grey traces to respective static red channel (mCherry) signals. Note that **g-h** show the same Y scale throughout, while **i** shows Y scales adjusted for each cell. All data are presented as mean ± SEM. ns: not significant. Source data are provided as a Source Data file.

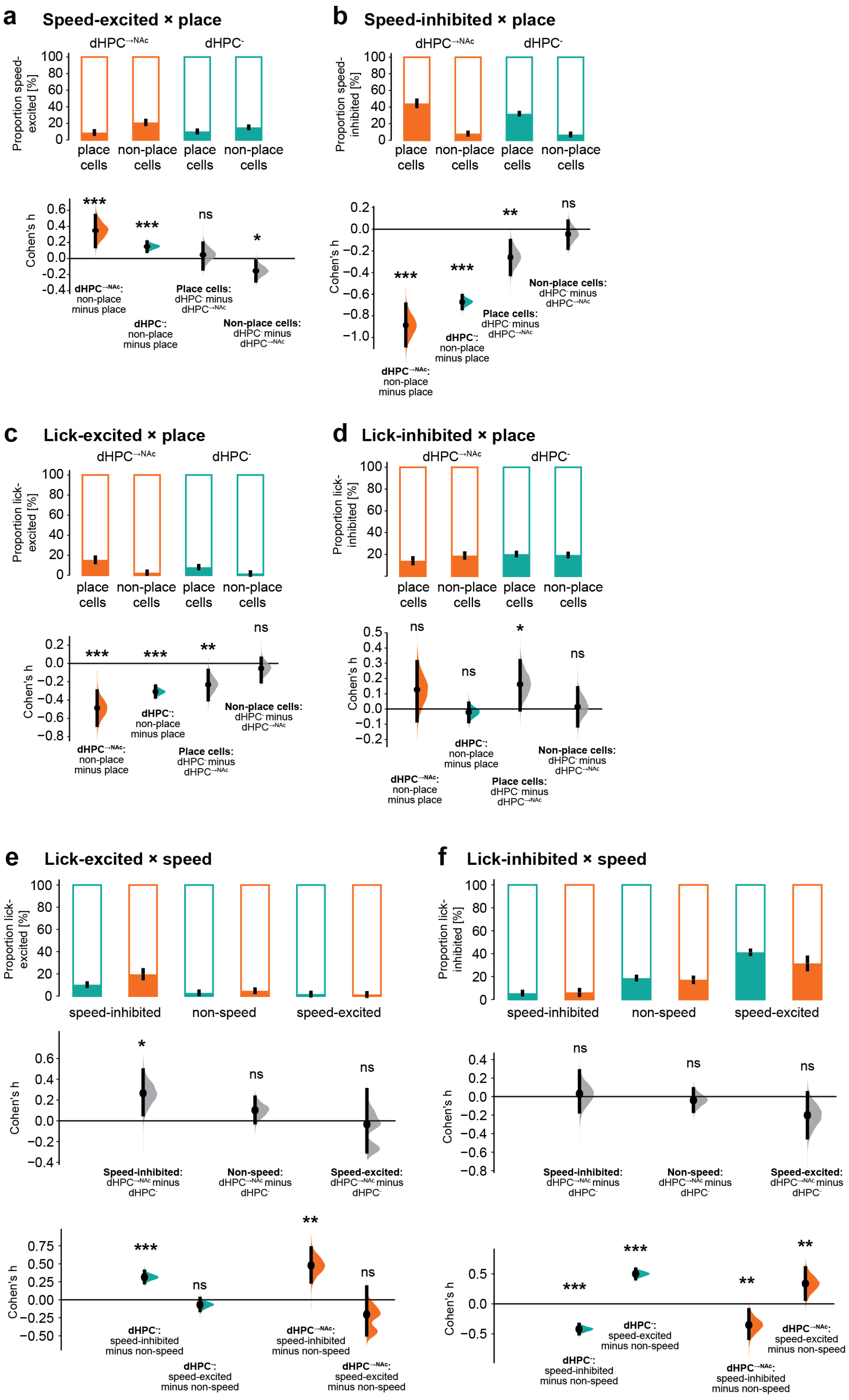

Fig. S10 | Enhanced conjunctive coding by dHPC$^{\rightarrow NAc}$ neurons.

Estimation statistics for Figs. 7b,d,f. **a,** Speed-excited neurons are overrepresented in non-place cells compared to place cells for dHPC$^{\rightarrow NAc}$ neurons (Cohen's *h* = 0.3493, 95% confidence

intervals: 0.1491 – 0.5369, *P* < 0.0001) and dHPC^{-} neurons (Cohen's *h* = 0.1495, 95% confidence intervals: 0.0898 – 0.2066, *P* < 0.0001). Proportions of speed-excited non-place cells are lower in dHPC^{-} neurons compared to dHPC$^{\rightarrow NAc}$ neurons (Cohen's *h* = -0.153, 95% confidence intervals: -0.2790 – -0.0309, *P* = 0.0106), but not for speed-excited place cells (Cohen's *h* = 0.0467, 95% confidence intervals: -0.1294 – 0.1909, *P* = 0.4978). **b,** Speed-inhibited neurons are overrepresented in place cells compared to non-place cells for dHPC$^{\rightarrow NAc}$ neurons (Cohen's *h* = -0.8846, 95% confidence intervals: -1.0742 – -0.6895, *P* < 0.0001) and dHPC^{-} neurons (Cohen's *h* = -0.6706, 95% confidence intervals: -0.7314 – -0.6120, *P* < 0.0001). Proportions of speed-inhibited place cells are higher in dHPC$^{\rightarrow NAc}$ neurons compared dHPC^{-} neurons (Cohen's *h* = -0.2582, 95% confidence intervals: -0.4177 – -0.1041, *P* = 0.0012), but not for speed-inhibited non-place cells (Cohen's *h* = -0.0442, 95% confidence intervals: -0.1760 – 0.0753, *P* = 0.4570). **c,** Lick-excited neurons are overrepresented in place cells compared to non-place cells for dHPC$^{\rightarrow NAc}$ neurons (Cohen's *h* = -0.4857, 95% confidence intervals: -0.6759 – -0.2996, *P* < 0.0001) and dHPC^{-} neurons (Cohen's *h* = -0.3084, 95% confidence intervals: -0.3667 – -0.2471, *P* < 0.0001). Proportions of lick-excited place cells are higher in dHPC$^{\rightarrow NAc}$ neurons compared to dHPC^{-} neurons (Cohen's *h* = -0.231, 95% confidence intervals: -0.3968 – -0.0763, *P* = 0.0070), but not for lick-excited non-place cells (Cohen's *h* = -0.0541, 95% confidence intervals: -0.2006 – 0.0571, *P* = 0.3842). **d,** Proportions of lick-inhibited neurons are not different between place cells compared to non-place cells for dHPC$^{\rightarrow NAc}$ neurons (Cohen's *h* = 0.1270, 95% confidence intervals: -0.0779 – 0.3108, *P* = 0.191) and dHPC^{-} neurons (Cohen's *h* = -0.0206, 95% confidence intervals: -0.0823 – 0.0383, *P* = 0.495). Proportions of lick-inhibited place cells are higher in dHPC^{-} neurons compared to dHPC$^{\rightarrow NAc}$ neurons (Cohen's *h* = 0.1621, 95% confidence intervals: -0.0059 – 0.3172, *P* = 0.0394), but not for lick-inhibited non-place cells (Cohen's *h* = -0.0145, 95% confidence intervals: -0.1105 – 0.1392, *P* = 0.8068). **e,** Proportions of lick-excited neurons are increased in speed-inhibited neurons compared to non-speed-tuned neurons for dHPC$^{\rightarrow NAc}$ populations (Cohen's *h* = 0.4778, 95% confidence intervals: 0.2461 – 0.7202, *P* = 0.0010) and dHPC^{-} populations (Cohen's *h* = 0.3144, 95% confidence intervals: 0.2382 – 0.3963, *P* < 0.0001). Proportions of lick-excited neurons are not different in speed-excited neurons compared to non-speed-tuned neurons for dHPC$^{\rightarrow NAc}$ populations (Cohen's *h* = -0.2045, 95% confidence intervals: -0.4881 – 0.1781, *P* = 0.0822) and dHPC^{-} populations (Cohen's *h* = -0.0685, 95% confidence intervals: -0.1491 – 0.0190, *P* = 0.0896). Proportions of lick-excited neurons among speed-inhibited neurons are increased in dHPC$^{\rightarrow NAc}$ neurons compared to dHPC^{-} neurons (Cohen's *h* = 0.2658, 95% confidence intervals: 0.0587 – 0.4896, *P* = 0.0104), but proportions of lick-excited neurons are not different for non-speed-tuned neurons (Cohen's *h* = 0.1024, 95% confidence intervals: -0.0185 – 0.2280, *P* = 0.1080) or speed-excited neurons (Cohen's *h* = -0.0336, 95% confidence intervals: -0.3002 – 0.2997, *P* = 0.6352). **f,** Proportions of lick-inhibited neurons are lower in speed-inhibited neurons compared to non-speed-tuned neurons for dHPC$^{\rightarrow NAc}$ populations (Cohen's *h* = -0.3514, 95% confidence intervals: -0.5755 – -0.0972, *P* = 0.0018) and dHPC^{-} populations (Cohen's *h* = -0.4214, 95% confidence intervals: -0.4986 – -0.3419, *P* < 0.0001). Proportions of lick-inhibited neurons are higher in speed-excited neurons compared to non-speed-tuned neurons for dHPC$^{\rightarrow NAc}$ populations (Cohen's *h* = 0.3379, 95% confidence intervals: - 0.0772 – 0.6008, *P* = 0.0094) and dHPC^{-} populations (Cohen's *h* = 0.4996, 95% confidence intervals: 0.4180 – 0.5774, *P* < 0.0001). Proportions of lick-inhibited neurons are not different between dHPC$^{\rightarrow NAc}$ neurons and dHPC^{-} neurons for speed-inhibited populations (Cohen's *h* = 0.0311, 95% confidence intervals: -

0.1650 – 0.2782, *P* = 0.6408), non-speed-tuned populations (Cohen's *h* = -0.0389, 95% confidence intervals: -0.1605 – 0.0848, *P* = 0.5106) or speed-excited neurons (Cohen's *h* = -0.2006, 95% confidence intervals: -0.4455 – 0.0412, *P* = 0.1020). Cumming estimation plots show the relative proportions of respectively tuned neurons for $dHPC^{-}$ (green) and $dHPC^{\rightarrow NAc}$ (red) populations, vertical bars representing s.d.; bottom panels show Cohen's *h* computed from 5,000 bootstrapped resamples (black dot, mean; black ticks, 95% confidence interval; filled curve, sampling-error distribution). ns: not significant, $^{*}P < 0.05$, $^{**}P < 0.01$, $^{***}P < 0.001$. Source data are provided as a Source Data file.

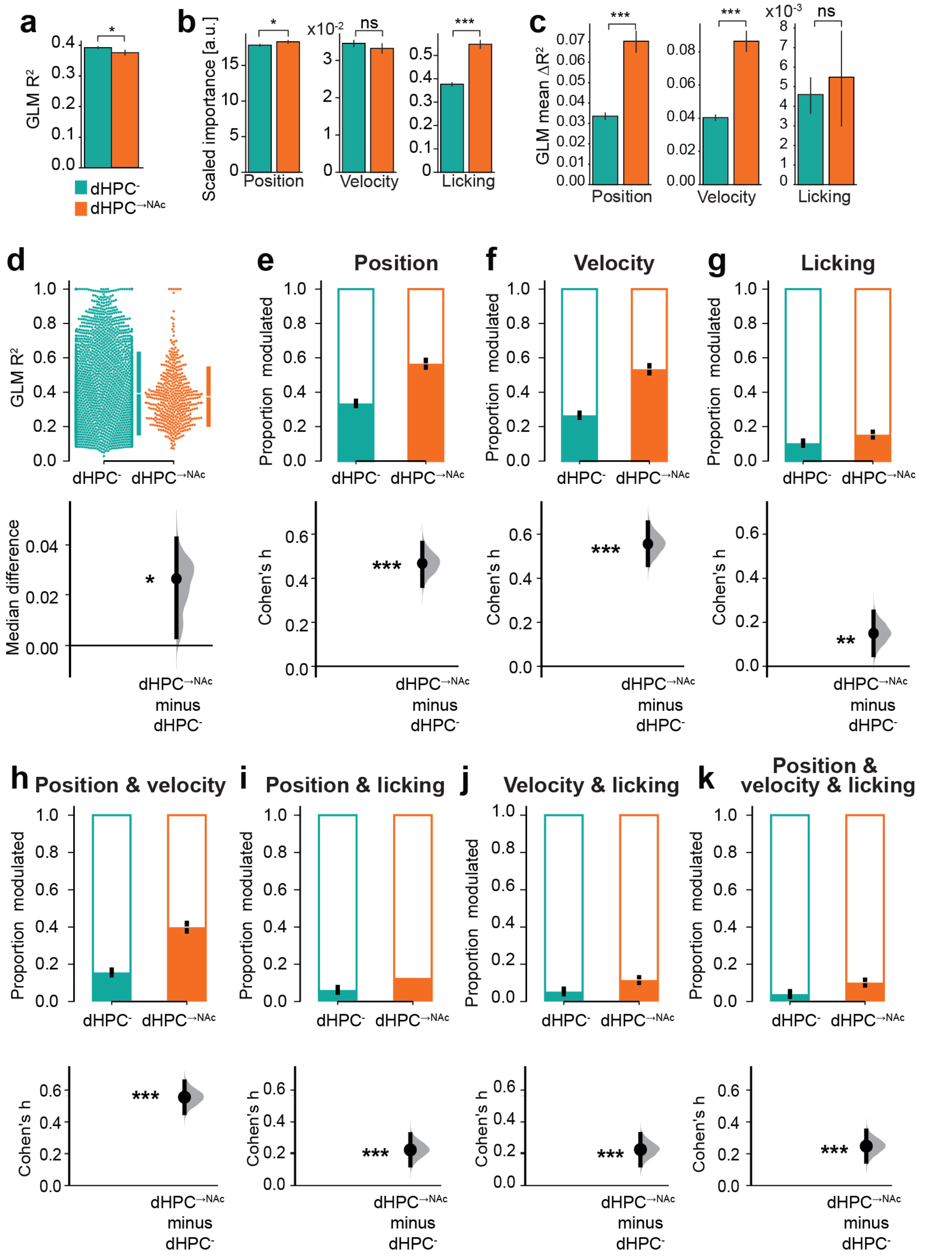

Fig. S11 | Generalized linear model reveals coding differences for dHPC→NAc neurons.

a, GLMs' explained variance R^2 is significantly lower for dHPC→NAc neurons (Welch's *t*(615.15) = 2.147, *P* = 0.032). **b,** Scaled feature importance (standardized GLM coefficients retrieved by *H2O*'s *varimp()* method) is higher in dHPC→NAc neurons for position (Welch's *t*(2508.09) = 2.162,

P = 0.031) and licking (Welch's *t*(1879.75) = 9.328, *P* < 0.001), but not for velocity (Welch's *t*(2609.15) = 0.881, *P* = 0.378). **c,** Mean R^2 differences between full and feature-shuffled models are significantly greater in dHPC$^{\rightarrow NAc}$ neurons for position (Welch's *t*(523.23) = 5.984, *P* < 0.001), velocity (Welch's *t*(497.69) = 7.704, *P* < 0.001), but not for licking (Welch's *t*(552.63) = 0.3626, *P* = 0.717). All data are presented as mean ± SEM. ns: not significant, **P* < 0.05, ****P* < 0.001. **d-k,** Estimation statistics for Figs. 8b-c, (**a**). **d,** GLM R^2 values of full models are higher in dHPC— neurons compared to dHPC$^{\rightarrow NAc}$ neurons, median difference = 0.0262 (95% confidence intervals: 0.0033, 0.0421), *P* = 0.0236. **e,** More dHPC$^{\rightarrow NAc}$ neurons are significantly modulated by position than dHPC— neurons, Cohen's *h* = 0.467687 (95% confidence intervals: 0.365854, 0.560568), *P* < 0.0001. **f,** More dHPC$^{\rightarrow NAc}$ neurons are significantly modulated by velocity than dHPC— neurons, Cohen's *h* = 0.555954 (95% confidence intervals: 0.460171, 0.653014), *P* < 0.0001. **g,** More dHPC$^{\rightarrow NAc}$ neurons are significantly modulated by licking than dHPC— neurons, Cohen's *h* = 0.149811 (95% confidence intervals: 0.051352, 0.24782), *P* = 0.0012. **h,** More dHPC$^{\rightarrow NAc}$ neurons are significantly modulated by both position and velocity than dHPC— neurons, Cohen's *h* = 0.555042 (95% confidence intervals: 0.456274, 0.653042), *P* < 0.0001. **i,** More dHPC$^{\rightarrow NAc}$ neurons are significantly modulated by both position and licking than dHPC— neurons, Cohen's *h* = 0.224136 (95% confidence intervals: 0.127388, 0.321765), *P* < 0.0001. **j,** More dHPC$^{\rightarrow NAc}$ neurons are significantly modulated by both velocity and licking than dHPC— neurons, Cohen's *h* = 0.226191 (95% confidence intervals: 0.127464, 0.322651), *P* < 0.0001. **k,** More dHPC$^{\rightarrow NAc}$ neurons are significantly modulated by both position, velocity, and licking than dHPC— neurons, Cohen's *h* = 0.247477 (95% confidence intervals: 0.150986, 0.344704), *P* < 0.0001. Cumming estimation plots show the raw data points (**d**) or relative proportions (**e-k**) of respectively tuned neurons for dHPC$^-$ (green) and dHPC$^{\rightarrow NAc}$ (red) populations, vertical bars representing s.d.; bottom panels show Cohen's *h* computed from 5,000 bootstrapped resamples (black dot, mean; black ticks, 95% confidence interval; filled curve, sampling-error distribution). ns: not significant, **P* < 0.05, ***P* < 0.01, ****P* < 0.001. Source data are provided as a Source Data file.

Supplementary Table 1, related to Figure 1i: 3-way ANOVA results of optogenetic inhibition effects on licking near a learned reward zone.

Effect	DFn	DFd	F	p	p<.05	ges
Group	1	6	2.84	0.143		0.133
Sex	1	6	0.0130	0.912		7.10E-04
Day	1	6	3.19	0.124		0.264
Group:Sex	1	6	3.78	0.1		0.170
Group:Day	1	6	0.596	0.469		0.0630
Sex:Day	1	6	5.25E-06	0.998		5.91E-07
Group:Sex:Day	1	6	0.0120	0.917		0.0010

DFn: degrees of freedom numerator (based on factor levels); DFd: degrees of freedom denominator (based on animals); ges: effect size generalized eta squared

Supplementary Table 2, related to Figure 1j: 3-way ANOVA results of optogenetic inhibition effects on learning to lick near a new reward zone.

Effect	DFn	DFd	F	p	p<.05	ges
Group	1	5	5.90E+00	0.059		0.393
Sex	1	5	1.55E+00	0.268		0.146
Day	1.1	5.52	1.83E+01	0.006	*	0.622
Group:Sex	1	5	1.34E-01	0.729		0.015
Group:Day	1.1	5.52	7.62E+00	0.034	*	0.407
Sex:Day	1.1	5.52	0.289	0.634		0.025
Group:Sex:Day	1.1	5.52	0.99	0.372		0.082

DFn: degrees of freedom numerator (based on factor levels); DFd: degrees of freedom denominator (based on animals); ges: effect size generalized eta squared

Supplementary Table 3, related to Figure 2f: 3-way ANOVA results of optogenetic activation on acute mouth motion.

Effect	DFn	DFd	F	p	p<.05	ges
Group	1	3	0.171	0.707		0.0540
Sex	1	3	2.38	0.221		0.441
Opto	1	3	0.228	0.017	*	0.0190
Group:Sex	1	3	0.0570	0.827		0.0180
Group:Opto	1	3	8.29	0.064		0.0070
Sex:Opto	1	3	0.266	0.641		2.25E-04
Group:Sex:Opto	1	3	0.0890	0.784		7.56E-05

DFn: degrees of freedom numerator (based on factor levels); DFd: degrees of freedom denominator (based on animals); ges: effect size generalized eta squared

Supplementary Table 4, related to Figure 3g-k: Mixed Linear Model Regression results of spatial tuning characteristics between projection subpopulations (fixed effect) across individual mice (random effect).

Outcome variable	Effect	*Beta*	*Std. Err.*	*z*	*p-value*	*[0.025*	*0.975]*
Spatial information	fixed	0.364	0.087	4.201	< 0.001	0.194	0.533
Spatial information	random	1.340	0.157	8.549	< 0.001	1.033	1.647
Sparsity	fixed	0.056	0.011	5.077	< 0.001	0.034	0.078
Sparsity	random	0.339	0.060	5.674	< 0.001	0.222	0.456
Reliability	fixed	-0.009	0.022	-0.391	0.696	-0.053	0.035
Reliability	random	0.504	0.044	11.336	< 0.001	0.416	0.591
Stability	fixed	0.011	0.014	0.739	0.460	-0.018	0.039
Stability	random	0.250	0.058	4.323	< 0.001	0.137	0.364
Δin-out place field	fixed	1.178	0.814	1.446	0.148	-0.418	2.774
Δin-out place field	random	10.706	1.123	9.535	< 0.001	8.506	12.907

Fixed effect: projection identity; random effect: mouse identity; p-value is not corrected for multiple comparisons

Supplementary Table 5, related to *Supplementary Figure 2g, Day 0*: 2-way ANOVA results on differential licking near reward zone.

Source	SS	DF	MS	F	*p-unc*	*np2*
Group	0.0271	1.0	0.027136	0.243799	0.655364	0.075159
Sex	0.0522	1.0	0.052221	0.469162	0.542549	0.135238
Group * Sex	0.0161	1.0	0.016061	0.144299	0.729341	0.045892
Residual	0.3339	3.0	0.111306			

SS: sums of squares; MS: mean squares; p-unc: uncorrected p-values; np2: partial eta-square effect sizes

Supplementary Table 6, related to *Supplementary Figure 2g, Day 1*: 2-way ANOVA results on differential licking near reward zone.

Source	SS	DF	MS	F	*p-unc*	*np2*
Group	0.0809	1.0	0.080869	3.978938	0.140040	0.570135
Sex	0.0246	1.0	0.024573	1.209033	0.351847	0.287247
Group * Sex	0.0052	1.0	0.005224	0.257057	0.647068	0.078923
Residual	0.0610	3.0	0.020324			

SS: sums of squares; MS: mean squares; p-unc: uncorrected p-values; np2: partial eta-square effect sizes

Supplementary Table 7, related to *Supplementary Figure 2g, Day 2*: 2-way ANOVA results on differential licking near reward zone.

Effect	DFn	DFd	F	p	p<.05	ges
Group	0.2125	1.0	0.212550	9.036332	0.057392	0.750755
Sex	0.0001	1.0	0.000074	0.003135	0.958870	0.001044
Group * Sex	0.0057	1.0	0.005687	0.241776	0.656656	0.074581
Residual	0.0706	3.0	0.023522			

SS: sums of squares; MS: mean squares; p-unc: uncorrected p-values; np2: partial eta-square effect sizes
