# Supplementary material for: A hippocampus-accumbens code guides goal-directed appetitive behavior
Source: Nat Commun. 2024 Apr 12;15:3196. doi: 10.1038/s41467-024-47361-x (PMC11015045; doi:10.1038/s41467-024-47361-x)
Supplement: Supplementary file 3 — Description of Additional Supplementary Files [file 41467_2024_47361_MOESM3_ESM.pdf]

Description of Additional Supplementary Files

Supplementary Video 1 | Optogenetic stimulation induces mouth movement. Five representative optogenetic stimulation trials from one experimental mouse expressing ChR2 and one control mouse expressing EYFP only. A light fiber in NAc stimulated excitatory hippocampal projections by shining 473 nm laser light at times indicated by blue squares (top) and blue traces (bottom). Optogenetic stimulation repeatedly induces lick behavior and a decrease in velocity in ChR2-expressing animals but not those expressing EYFP. Playback speed equals original speed.

Supplementary Video 2 | In vivo dual-color two-photon calcium imaging of a mouse during goal-directed navigation. Representative example of dual-color twophoton calcium imaging combined with goaldirected navigation and lick behavior. Infrared camera images show face (top left) and body (top right) tracking. Calcium activity is shown by overlaying denoised GCaMP6s activity (green) onto average mCherry signal (red; bottom left); traces represent denoised GCaMP6s activity of mCherry-negative (green) and mCherry-positive (red; NAc-projecting) hippocampal neurons (bottom right). Playback speed is 2X original speed.

Supplementary Video 3 | Appetitive lickexcited calcium dynamics. Representative example of dual-color twophoton calcium imaging during lick behavior. Shown is motion-corrected (rolling average of 10 frames) but otherwise raw imaging data of the same recording shown in Figure 5, highlighting lick-excited dHPC neurons. Left side (green) shows dynamic calcium signals collected from GCaMP6s, right side (red) shows static mCherry signal. Polygons drawn correspond to deconvolved calcium traces below. Licking trace shows normalised lick spout signal, and reward trace shows reward dispensation. Playback speed is 2X original speed.
