# Supplementary material for: A hippocampus-accumbens code guides goal-directed appetitive behavior
Source: Nat Commun. 2024 Apr 12;15:3196. doi: 10.1038/s41467-024-47361-x (PMC11015045; doi:10.1038/s41467-024-47361-x)
Supplement: Supplementary file 7 — Reporting Summary [file 41467_2024_47361_MOESM7_ESM.pdf]

nature portfolio

Corresponding author(s): Oliver Barnstedt and Stefan Remy

Last updated by author(s): 21/02/2024

Reporting Summary

Nature Portfolio wishes to improve the reproducibility of the work that we publish. This form provides structure for consistency and transparency in reporting. For further information on Nature Portfolio policies, see our Editorial Policies and the Editorial Policy Checklist.

Statistics

For all statistical analyses, confirm that the following items are present in the figure legend, table legend, main text, or Methods section.

n/a	Confirmed	
☐	☒	The exact sample size (*n*) for each experimental group/condition, given as a discrete number and unit of measurement
☐	☒	A statement on whether measurements were taken from distinct samples or whether the same sample was measured repeatedly
☐	☒	The statistical test(s) used AND whether they are one- or two-sided<br>*Only common tests should be described solely by name; describe more complex techniques in the Methods section.*
☐	☒	A description of all covariates tested
☐	☒	A description of any assumptions or corrections, such as tests of normality and adjustment for multiple comparisons
☐	☒	A full description of the statistical parameters including central tendency (e.g. means) or other basic estimates (e.g. regression coefficient) AND variation (e.g. standard deviation) or associated estimates of uncertainty (e.g. confidence intervals)
☐	☒	For null hypothesis testing, the test statistic (e.g. *F*, *t*, *r*) with confidence intervals, effect sizes, degrees of freedom and *P* value noted<br>*Give P values as exact values whenever suitable.*
☒	☐	For Bayesian analysis, information on the choice of priors and Markov chain Monte Carlo settings
☒	☐	For hierarchical and complex designs, identification of the appropriate level for tests and full reporting of outcomes
☐	☒	Estimates of effect sizes (e.g. Cohen's *d*, Pearson's *r*), indicating how they were calculated

Our web collection on statistics for biologists contains articles on many of the points above.

Software and code

Policy information about availability of computer code

Data collection	Inspector software (LaVision BioTec) was used for 2-photon microscope control and image acquisition. Pylon Camera Software Suite (Basler) was used for behavioral tracking camera recordings. Virtual reality control and analog signal data collection was performed using Python 2.7 with packages PyDAQmx 1.2.3, PyOgre, and Openpyxl 2.4.0.
Data analysis	Behavioral data were analyzed using DeepGraphPose/DeepLabCut 2.0. Calcium signals were processed using CaImAn 1.6.2. GLM was built using Python's H2O 3.18. 3-way ANOVAs were calculated with R 4.3.1 and package rstatix. All further analyses were performed using Python 3.10.4 and packages Pandas 1.4.3, Pingouin 0.5.2, Numpy 1.22.3, Scipy 1.7.3, Matplotlib 3.5.1, Seaborn 0.11.2, Nptdms 1.4.0, Numba 0.55.2, OpenCV 4.5.5, Tifffile 2022.5.4, Dabest 2023.2.14. All code to generate main figures has been deposited under https://github.com/obarnstedt/dHPC-NAc.

For manuscripts utilizing custom algorithms or software that are central to the research but not yet described in published literature, software must be made available to editors and reviewers. We strongly encourage code deposition in a community repository (e.g. GitHub). See the Nature Portfolio guidelines for submitting code & software for further information.

Data

Policy information about availability of data

All manuscripts must include a data availability statement. This statement should provide the following information, where applicable:

- Accession codes, unique identifiers, or web links for publicly available datasets
- A description of any restrictions on data availability
- For clinical datasets or third party data, please ensure that the statement adheres to our policy

The raw and processed data generated in this study have been deposited on Zenodo under DOI https://doi.org/10.5281/zenodo.10698565. The data used to generate all main and supplementary figures are provided in the Source Data file.

Research involving human participants, their data, or biological material

Policy information about studies with human participants or human data. See also policy information about sex, gender (identity/presentation), and sexual orientation and race, ethnicity and racism.

Reporting on sex and gender	N/A
Reporting on race, ethnicity, or other socially relevant groupings	N/A
Population characteristics	N/A
Recruitment	N/A
Ethics oversight	N/A

Note that full information on the approval of the study protocol must also be provided in the manuscript.

Field-specific reporting

Please select the one below that is the best fit for your research. If you are not sure, read the appropriate sections before making your selection.

☒ Life sciences ☐ Behavioural & social sciences ☐ Ecological, evolutionary & environmental sciences

For a reference copy of the document with all sections, see nature.com/documents/nr-reporting-summary-flat.pdf

Life sciences study design

All studies must disclose on these points even when the disclosure is negative.

Sample size	The dataset includes n=5,372 hippocampal cells including n=444 mCherry-positive ones putatively projecting into NAc, collected over a total of 19 imaging sessions in 6 mice. A total of 18 mice were used in the spatial learning experiments, including the 6 mice we collected imaging data from. Optogenetic activation experiments are based on 4 mice expressing ChR2 and 3 mice expressing EYFP control. Optogenetic inhibition experiments are based on 5 mice expressing ArchT and 5 mice expressing EGFP control. No statistical methods were used to pre-determine sample sizes but our sample sizes are similar to those reported in previous publications using two-photon calcium imaging of hippocampal pyramidal neurons (e.g. Terada et al., 2022, Nature; Hainmueller & Bartos, 2018, Nature) and similar optogenetic studies (Jiang et al., 2022, Nature Neuroscience; Park et al., 2016, eLife).
Data exclusions	One EGFP control mouse was excluded from day 6 of inhibition experiments onwards due to technical problems. Inclusion criteria for spatiotemporal components were based on blind adaptive thresholding of CaImAn's quality values of 1) signal-to-noise ratio, 2) convolutional neural network classifier similarity, and 3) spatial correlation r. Reward zone decoding comparing (non-)projecting neurons was performed only with recordings containing >10 projection neurons.
Replication	Behavioral performance in our head-fixed spatial reward learning task were replicated across three independent mouse cohorts (C57BL/6J mice and Thy1-GCaMP6s mice). For imaging and optogenetic experiments, data were collected across multiple days across multiple biological replicates. Main effects were consistent across individual mice and cells within each group, as evident by the presentation of individual data throughout the paper.
Randomization	C57BL/6J mice were randomly allocated into experimental (injected with ChR2/ArchT virus) and control (injected with EYFP/EGFP-only virus) groups by an experimenter not involved in behavioral testing.
Blinding	Optogenetic behavioral experiments were performed by an experimenter blinded to the experimental group. Spatiotemporal component curation was performed by an experimenter blinded to cellular identity or behavioral correlates.

Reporting for specific materials, systems and methods

We require information from authors about some types of materials, experimental systems and methods used in many studies. Here, indicate whether each material, system or method listed is relevant to your study. If you are not sure if a list item applies to your research, read the appropriate section before selecting a response.

Materials & experimental systems

n/a	Involved in the study
☒	☐ Antibodies
☒	☐ Eukaryotic cell lines
☒	☐ Palaeontology and archaeology
☐	☒ Animals and other organisms
☒	☐ Clinical data
☒	☐ Dual use research of concern
☒	☐ Plants

Methods

n/a	Involved in the study
☒	☐ ChIP-seq
☒	☐ Flow cytometry
☒	☐ MRI-based neuroimaging

Animals and other research organisms

Policy information about studies involving animals; ARRIVE guidelines recommended for reporting animal research, and Sex and Gender in Research

Laboratory animals	These experiments used adult male and female C57BL/6J mice (Charles River Laboratories, UK) or transgenic Thy1-GCaMP6s mice (The Jackson Laboratories; C57BL/6J-Tg(Thy1-GCaMP6s)GP4.3Dkim/J, stock number 024275 RRID: IMSR_JAX:024275; maintained on a C57BL/6J background). Mice were housed with 1-5 littermates per cage at 19–23°C ambient temperature and 40–70% humidity. Mice were put under food restriction and a reversed 12/12h light/dark cycle before the beginning of habituation for the duration of experiments. Mice were 3-6 months old at the time of testing.
Wild animals	No wild animals were used in the study.
Reporting on sex	Behavioral and optogenetic experiments were carried out in female and male mice; sex-specific single data points are shown throughout the manuscript; ANOVA tests showed no significant sex-dependent differences. Imaging experiments were performed only in male mice.
Field-collected samples	No field collected samples were used in the study.
Ethics oversight	All experiments were performed according to the Directive of the European Communities Parliament and Council on the protection of animals used for scientific purposes (2010/63/EU) and were approved by the animal care committee of North Rhine-Westphalia, Germany.

Note that full information on the approval of the study protocol must also be provided in the manuscript.
